# Occupational Exposure of Shiraz Dental Students to Patients’ Blood and Body Fluid

**Published:** 2015-09

**Authors:** Soheila Shaghaghian, Ali Golkari, Soheil Pardis, Ali Rezayi

**Affiliations:** 1 Dept. of Dental Public Heath, School of Dentistry, Shiraz University of Medical Sciences, Shiraz, Iran.; 2 Dept. of Oral and Maxillofacial Pathology, School of Dentistry, Shiraz University of Medical Sciences, Shiraz, Iran.; 3 Undergraduate Student, School of Dentistry, Shiraz University of Medical Sciences, Shiraz, Iran.

**Keywords:** Occupational Exposure, Blood, Body Fluids, Dental Students

## Abstract

**Statement of the Problem:**

Exposure to patients’ blood and body fluids would prone the dental students to the risk of blood borne infections. Several studies have shown a high prevalence of these exposures in dental settings particularly in developing countries. However, few studies have evaluated the epidemiology of these exposures in dental students in Iran.

**Purpose:**

To assess the epidemiology of occupational exposures among dental students and consequently designing the appropriate interventions in order to prevent these exposures.

**Materials and Method:**

In this cross-sectional study performed during March to June 2011, all 191 Shiraz clinical dental students were asked to complete a self-administered questionnaire. This questionnaire included demographic information and experience of sharp injuries and mucocutaneous contaminations. Chi square and t-test were employed to evaluate the risk factors of exposure.

**Results:**

80%of the participants were exposed to the patients’ blood or body fluids during their clinical course. No association was found between the exposure and demographic factors. Injection needle and recapping were the most common causes of these injuries. The most common sites that were injured and caused mucocutaneous contamination were finger and face, respectively. The most frequent activity causing contamination was using high-speed rotary instruments. Only 6.4% of the exposures had been reported to the related authorities and the remains were underreported.

**Conclusion:**

Blood and body fluid exposure in dental setting is common and a lot of them are not reported. To reduce the hazards of these exposures, infection control authorities should design interventions especially for mentioned high-risk conditions. They should change dental students’ behavior especially regarding not recapping injection needles and using eyewear. Dental schools seem to need a management center and a standard protocol for following up the exposures.

## Introduction

Occupational exposure is defined as cutaneous injury with contaminated sharp instruments such as needles (sharp injury), or contamination of skin or mucosa to patients’ blood, saliva or other potentially infectious body fluids (mucocutaneous contamination).[1] These exposures are one of the occult problems of the health care workers (HCWs) which unfortunately are not uncommon.[2] It is estimated that more than 600,000 sharp injuries and 200,000 blood and body fluids (BBF) contaminations occur annually in the United States.[3] Among the HCWs, dental practitioners are more prone to the exposures due to close contact with the patients' oral cavity, frequent use of sharp instruments and working with high-speed rotary instruments that produce contaminated aerosols.[4] In a study conducted in Washington, 20% of occupational injuries were in the dental profession.[5] In Askarian *et al.* study (2004) 73.7% of Shiraz dental students were injured by a sharp instrument at least once.[2]

These exposures predispose the HCWs to more than 20 microorganisms that cause blood borne infections in which hepatitis B virus (HBV), hepatitis C virus (HCV) and human immunodeficiency virus (HIV) are reported to be the most common.[6] Reports from WHO in 2002 revealed that 2.5% of HIV and 40% of HBV and HCV infections in HCWs worldwide were caused by occupational exposures.[7] Other reports showed 319 cases of HIV infection worldwide that were caused by occupational injuries (102 definite and 217 probable cases), of which nine cases are pertained to the dental HCWs.[8] In Iran, detection of 24290 HIV^+^ cases[9] and estimation of 3% HBV^+^ and 3-5% HCV^+^ carriers in the population;[10] highlights the importance of occupational exposures.

Despite the post exposure prophylactic measures for HBV and HIV, very small proportion of these exposures are reported. British studies estimate that unreported sharp injuries may be ten times more frequent than the reported cases.[11] In two study conducted in Iran, 85% and 90% of students did not report their injuries.[2, 12] Because of few numbers of reports, post exposure prophylactic management is not possible in lots of cases. This highlights the importance of interventions to prevent sharp injuries and improve reporting system. In order to plan these interventions in dental schools, some epidemiologic data are required to find high-risk groups and procedures to focus on. To the best of our knowledge, there are not any available researches evaluating the epidemiology of dental occupational exposures in Iran up to the time of this study. We hope the results of this study help infection control officials in designing interventions to prevent these exposures.

## Materials and Method

This cross-sectional survey was conducted at Shiraz University of Medical Sciences (one of the biggest universities of Iran) by distribution of questionnaires among all 191 clinical dental students from March to June, 2011(consensus).

The questionnaire was prepared by incorporating the questions extracted from questionnaires of other studies[2, 4, 12-14] and then revised by a group of students, professors and the members of infection control committee of dental school who confirmed its content validity. This questionnaire consists of three parts:

1. The students' demographic information and the number of sharp injuries and mucocutaneous contaminations (six questions)
2. Characteristics of the sharp injuries (ten questions)
3. Characteristics of the mucocutaneous contaminations (11 questions)

The questionnaires were filled by interviewing all participants. A dental student who was adequately trained regarding the issues of occupational exposure and the contents of the questionnaire conducted the interviews. Before interviewing, the participants were informed about the purpose of the study and assured about the confidentiality of their information and asked for verbal consent.

The collected data were analyzed by SPSS 17. Standard descriptive statistical techniques were used for determination of sharp injury and mucocutaneous contamination prevalence. Association between independent variables and exposure were evaluated by Chi square and independent sample student t-test. An alpha level of 0.05 was used to determine statistical significance.

Ethical issues were considered during each step of the research process .The study was limited to the students who consented to participation .The questionnaires did not include any personal identification and the access to the data was restricted to the research team. 

## Results

All of the 191 clinical dental students who were recruited in our study, filled in the questionnaire (response rate = 100%). The mean age was 24.8 years and 55.5% were female (Table 1). Eighty-three students (43.5%) had been injured with sharp objects and 122 students (63.9%) had contaminated with the patients' BBF during their clinical experience. Only 37 students (19.4%) had not encountered either kind of the above exposures. There is no significant statistical difference between the exposed and non-exposed students to the patients' BBF regarding age (*p*= 0.38), educational level (*p*= 0.55) and sex (*p*= 0.57) (Table 1).

**Table 1:** Clinical students of Shiraz dental school based on occupational exposure (N=191)

**Characteristics**	**All students**	**Exposed students**	**Non-exposed students**	**p value**
Age (Y) mean ±SD	24.8±6.7	24.6±6.3	25.8±8.1	0.38*
Educational level N(%) 4th grade 5th grade 6th grade	53(27.7) 66(34.6) 72(37.7)	42(79.2) 56(84.8) 56(77.8)	11(20.8) 10(15.2) 16(22.2)	0.55**
Sex N(%) Female Male	106(55.5) 85(44.5)	87(82.1) 67(78.8)	19(17.9) 18(21.2)	0.57**

N= number Y= year SD= standard deviation             *Independent sample student t-test was used     ** Chi square test was used

A total of 103sharp injuries had occurred in 83 out of 191 dental students. Sixty-five students (78%) experienced sharp injury only once and 18 students (22%) experienced it two or three times. The most common body area, instrument and procedure regarding sharp injury was finger (91.3%), injection needle (38.8%) and needle recapping (19.4%) respectively. The department in which most of the injuries occurred was the oral and maxillofacial surgery department (Table 2).

**Table 2:** Epidemiologic characteristics of occupational exposures in Shiraz dental students

**Sharp Injuries (N=103)**		**Mucocutaneous Contamination (N=207)**	
**Characteristics**	**N(%)**	**Characteristics**	**N(%)**
Location Finger Hand	94(91.3) 9(8.7)	Location Face Hand Eye	173(83.6) 27(13) 7(3.4)
Instrument Injection needle Endodontic file Laboratory knife Dental probe Elevator Suture needle Orthodontic wire Scaling instrument	40(38.8) 25(24.3) 14(13.6) 7(6.8) 7(6.8) 5(4.9) 3(2.9) 2(1.9)	Body fluid Saliva Blood	205(99) 2(1)
Activity Recapping Injection Dental lab procedures Procedures in phantom head preclinic Surgery Suturing Dental procedure aiding Injury with left away objects Others	20(19.4) 14(13.6) 14(13.6) 11(10.7) 8(7.8) 4(3.9) 2(1.9) 1(1) 29(28.1)	Activity Working with rotary instruments Scaling Surgery Taking impressions Observation of procedures Patients' vomiting, coughing or sneezing Contact with contaminated objects	96(46.4) 41(19.8) 35(16.9) 21(10.2) 9(4.3) 5(2.4) 0(0)
Dental school department Surgery Laboratory Endodontics Periodontics Pedodontics Restorative dentistry Removable prosthodontics Fixed prosthodontics Orthodontics General dentistry	41(39.8) 26(25.2) 15(14.6) 7(6.8) 5(4.8) 4(3.9) 3(2.9) 1(1) 1(1) 0(0)	Dental school department Periodontics Restorative dentistry Surgery Removable prosthodontics Pedodontics Fixed prosthodontics Endodontics General dentistry Radiology	48(23.2) 44(21.3) 42(20.3) 20(9.6) 18(8.7) 15(7.2) 13(6.3) 5(2.4) 2(1)
N= number			

Of the 191 students participated in our study, 122 students reported 207 times of BBF contamination. Fifty-eight students (47.5%) experienced BBF contamination only once and 64 students (52.5%) experienced it two to four times. Saliva was the most common fluid causing the contamination (99%) and blood was the source of contamination of only two cases (1%). Facial skin was the most common contaminated area (83.6%). The most common activity causing mucocutaneous contamination was working with high speed rotary instruments (46.4%) (Table 2).

It is worth mentioning that none of these exposed students mentioned a cutaneous lesion (ulceration, erosion, dermatitis) on the contaminated area. Furthermore, they did not identify any of the source patients of exposure as known case of hepatitis and acquired immunodeficiency syndrome (AIDS) or the high risk groups for these diseases including repeated receivers of blood products (hemophilia, thalassemia), intra venous drug abusers, hemodialysis and multisexual partner patients.

Epidemiologic characteristics of the departments of the dental school regarding BBF exposure of the students are summarized in Table 3. The most common area of the body involved by sharp injuries in all of the departments was the finger. Needle recapping was the most common cause of sharp injury in the restorative dentistry, pedodontics and surgery departments. Mucocutaneous contamination with blood occurred only once in removable prosthodontics department during impression procedures and once in surgery department during surgery observation. The most common contaminated area in most departments was the face. Seven cases of eye contamination with saliva occurred in periodontics (two cases), fixed prosthodontics (one case), surgery (three cases) and pedodontics (one case) departments. Two of these cases occurred during working with high-speed rotary instruments, three cases during surgery and two cases during scaling. Working with high-speed rotary instruments was the most common activity causing mucocutaneous contamination in the most departments. However, scaling was the most common procedure causing mucocutaneous contamination in periodontics department, so was about the impression procedure in removable prosthodontics and coughing, sneezing and vomiting in radiology departments.

**Table 3:** Epidemiologic characteristics of Shiraz dental school departments regarding occupational exposures

**Department**	**Sharp injuries (N=103)**			**Mucocutaneous contaminations (N=207)**		
	**Instrument (N)**	**Location** **(N)**	**Activity (N)**	**Body fluid** **(N)**	**Location** **(N)**	**Activity (N)**
Periodontics	Dental probe (5) Scale (2)	Finger (6) Hand (1)	Non-surgical treatments (7)	Saliva(48)	Face (44) Hand (2) Eye (2)	Scaling (41) Working with rotary instruments (5) During observation (1) Patient's coughing, sneezing or vomiting (1)
Endodontics	Endodontic file (15)	Finger (15)	Non-surgical treatments (15)	Saliva(13)	Face (13)	Working with rotary instruments (13)
Restorative dentistry	Injection needle (4)	Finger (4)	Recapping (3) Injection (1)	Saliva(44)	Face (44)	Working with rotary instruments (41) During observation (2) Patient's coughing, sneezing or vomiting(1)
Fixed prosthodontics	Dental explorer (1)	Finger (1)	Non-surgical treatments (1)	Saliva(15)	Face (12) Hand (2) Eye (1)	Working with rotary instruments (13) Taking impression (2)
Removable prosthodontics	Laboratory knife (3)	Finger (3)	Non-surgical treatments (3)	Saliva(19) Blood (1)	Hand (17) Face (3)	Taking impression (19) Patient's coughing, sneezing or vomiting (1)
Surgery	Injection needle (29) Elevator (7) Suture needle (5)	Finger (38) Hand (3)	Recapping(14) Injection (13) Surgery (8) Suturing (4) Surgery aiding (2)	Saliva(41) Blood (1)	Face (34) Hand (5) Eye (3)	Surgery (34) During observation (7) Working with rotary instruments (1)
Laboratory	Laboratory knife (11) Endodontic file (10) Injection needle (2) Orthodontic wire (2) Dental explorer (1)	Finger (21) Hand (5)	Denture processing (12) during procedures in phantom head lab (11) Non-surgical treatments (2) injury with left-over instrument (1)			
Pedodontics	Inection needle (5)	Finger (5)	Recapping (3) Non-surgical treatments (2)	Saliva(18)	Face (17) Eye (1)	Working with rotary instruments (18)
Orthodontics	Orthodontic wire (1)	Finger (1)	Non-surgical treatments (1)			
General dentistry				Saliva(5)	Face (4) Hand (1)	Working with rotary instruments (4) Surgery (1)
Radiology				Saliva(2)	Face (2)	Patient's coughing, sneezing or vomiting (2)

N= number

Only 14.6% of injured cases (15/103) and 2.4% of mucocutaneous contaminated cases (5/207) were reported. However, none of the two cases of blood contamination was reported and only two cases of eye contamination were testified. BBF exposures, occurring in surgery department, were reported more frequently than the exposures occurring in non-surgery departments (12% vs. 4.4%, *p*= 0.015).

Only ten (9.7%) cases of sharp injury and two (0.96%) cases of mucocutaneous contamination had undergone follow up measures respectively. However, none of them had received anti-retrovirus drug or hepatitis B Immunoglobulin. 

## Discussion

Only 19% of the 191 dental students, participated in this study, did report “no exposure” to patients' BBF. This high prevalence of occupational exposure is comparable with many similar studies[4, 12-13,15-17] both in Iran and in other parts of the world. Moreover, the highest number of sharp injuries was happened by injection needles, which has been reported as the major cause of percutaneous injuries among dental professionals in several other studies[4-5,18-19] In our study and several other studies,[14, 19-20] especially in the Middle East, needle recapping was reported to be the most common procedure associated with injuries in dentistry. However, in a study conducted in Brazil, needle recapping was only the injury cause of 3.5% of students.[17] There is good evidence that sharp injuries could be reduced by up to 70% if recapping was avoided.[21] This evidence clearly indicates the necessity of further interventions for safely discarding dental needles among Iranian dental health care. Education on how to avoid needling including not recapping has broadly been provided to dentists and dental students. However, our result shows that the education has not been sufficient. Dental students need more education about prompt disposal of needles or use of single scoop techniques. Furthermore, interventions should be headed towards provision of safety-enhanced devices and disposable dental syringes in dental settings.

In the current study, similar to other studies,[14, 22] more injuries have occurred in the surgery department than other departments. This could be explained by the inherent nature of the procedures done in this department. At the same time, if well-trained, the greatest decrease in the number of BBF exposures among dental students could be achieved in the surgery ward.

Most contaminations to patients' fluids in this study were recorded to be with saliva rather than blood. However, in dental practice, saliva is recognized as a potentially infectious material due to the possible existence of invisible blood in it.[1] The mixture of small amounts of blood with saliva is more important when using rotary instruments and the results of the current study indicate that most contaminations with saliva occur when using these instruments.

The results show that dental students' faces were exposed to patients' BBF in a considerable number of occasions. Eyes were also exposed in seven occasions. Dental procedures generally produce splatters and aerosols. Therefore, dentists should routinely use personal protective equipment such as facemask, eyewear, and preferably face shield. Although the importance of using proper protective equipment is well established,[17] only 52-62% of dentists have routinely used appropriate equipment to protect their faces.[2, 4, 12] One of the most important reasons that dentists did not use such equipments routinely was their unclear view, fog and reflection when using them.[4] Therefore, clear, non-fog and non-reflective protective eyewear should be easily available to dental team. Moreover, effective educational programs about standard precautions and using personal protective equipment are desirable.

The exposed students did not recognize any of the source patients as carrier of HBV, HCV or HIV or belonging to high-risk groups of acquiring these microorganisms. This finding differs from similar studies.[5, 14-15,20] This difference is probably because Iranian dentists did not try enough to recognize carrier and high risk groups of these diseases. It should be reminded that there are more than 24000 known HIV^+^ cases in the country[9] and 3% and 3-5% of individuals in our population are carriers of HBV and HCV, respectively.[10] Therefore, dental students should be well educated about the importance of careful evaluation of the source patients.

Low compliance on reporting occupational exposures in this study and also in several other studies [4, 12-13, 16 , 23-24] is another problem. Maybe, students’ believes are the reasons for not reporting. They believe that nobody is indicated to receive the exposure report[23] and these kinds of exposures do not have a significant risk.[25] Moreover, they think the reporting is time consuming,[25] not necessary,[23] and would not influence the outcome.[12] Furthermore, most students do not know how, where, and to whom exposures should be reported.[12] Even if the students know that they should report such exposures, high workloads may prevent them to report.[25] However, the main reason is very simple in Iran; the students do not know that all injuries should be reported.[12] Therefore, it seems necessary to develop a standard reporting protocol for dental schools in Iran, to set up a management center for following up the exposed students and sources of exposures, and to educate dental students about the possibilities of prophylaxis after BBF exposure. In Shiraz Dental School, Infection Control Committee manages and follows the exposed students. However, the results of this study highlight the need for similar centers in other cities. Furthermore, appropriate approach should be adopted to level up the students’ attitudes towards reporting exposures. These interventions should lead not only to more reporting the exposure but also to better compliance with post exposure prophylaxis. The latter is very important since in our study; similar to other studies,[14, 23, 25] a few of exposed dental HCWs had compliance with necessary actions.

Stating that no relationship was found between exposure and demographic factors in this study; there is no need to focus on specific groups when planning interventions.

 In this study, all clinical students of Shiraz Dental School completed the questionnaire, in a stress-free situation with adequate time, ensuring their confidentiality. Therefore, relatively accurate results are reported. However, the potential memory limitations associated with retrospective data collection applies to this study as well. To be more accurate, data on exposures should be recorded routinely in shorter periods in a designed center and in time. 

## Conclusion

This study highlighted the high-risk circumstances in dental occupational exposures. Infection control authorities have an important role in designing appropriate strategies to reduce the hazard of these situations. Our study recommends:

- More emphasis and encouragement on not recapping the injection needles.
- Making available safety enhanced needles in dental settings especially in surgical wards.
- Emphasizing on the potentially infectious nature of saliva in dental procedures.
- Promoting compliance with standard precautions especially using protective eyewear, facemask and face shield.
- Developing a standard reporting protocol for dental schools and setting up a management center for following the exposed personnel.
- Changing the attitude of dental students about the importance of reporting all exposures and role of post exposure prophylaxis in preventing the transmission of blood borne pathogens.
